# Long-Term Outcomes among Kidney Transplant Recipients and after Graft Failure: A Single-Center Cohort Study in Brazil

**DOI:** 10.1155/2019/7105084

**Published:** 2019-04-02

**Authors:** Paula Rebello Bicalho, Lúcio R. Requião-Moura, Érika Ferraz Arruda, Rogerio Chinen, Luciana Mello, Ana Paula F. Bertocchi, Erika Lamkowski Naka, Eduardo José Tonato, Alvaro Pacheco-Silva

**Affiliations:** ^1^Renal Transplantation Division, Hospital Israelita Albert Einstein, São Paulo, Brazil; ^2^Medicine School, Universidade Nove de Julho, São Paulo, Brazil; ^3^Department of Medicine, Nephrology Division, Universidade Federal de São Paulo, Brazil

## Abstract

**Background:**

The results of kidney transplantation are impacted by the categories of events responsible for patient death and graft failure. The objective of this study was to evaluate the causes of death and graft failure and outcomes after graft failure among kidney transplant recipients.

**Methodology:**

A retrospective cohort study was conducted with 944 patients who underwent kidney transplantation. Outcomes were categorized in a managed and hierarchical manner.

**Results:**

The crude mortality rate was 10.8% (n=102): in 35.3% cause of death was infection, in 30.4% cardiovascular disease, and in 15.7% neoplasia and in 6.8%, it was not possible to determine the cause of death. The rate of graft loss was 10.6%. The main causes of graft failure were chronic rejection (40%), acute rejection (18.3%), thrombosis (17.3%), and recurrence of primary disease (16.5%). Failures due to an acute rejection occurred earlier than those due to chronic rejection and recurrence (*p*<0.0001). As late causes of graft loss, death with the functioning kidney occurred earlier than recurrence and chronic rejection (*p*=0.008). The outcomes after graft failure were retransplantation in 26.1% and death in 21.4%, at a mean of 25.5 and 21.4 months, respectively.

**Conclusion:**

It was possible to identify more than 90% of the events responsible for the deaths of transplanted patients, predominantly infectious and cardiovascular diseases. Among the causes of graft failure, chronic and acute rejections and recurrence were the main causes of graft failure which were followed more frequently by retransplantation than by death on dialysis.

## 1. Introduction

Renal transplantation is the treatment of choice for patients with advanced kidney disease, even when compared with more sophisticated dialysis modalities [1–4]. In recent decades, there has been a substantial improvement in adverse outcomes related to renal transplantation in the short term, such as the incidence of acute rejection [5] and better management of delayed graft function [6, 7], but a proportional improvement in long-term outcomes was not observed, even in the most recent eras [8]. According to previous studies, the main causes of renal graft loss are chronic rejection, death with the functioning kidney, recurrence of the underlying disease, and acute rejection [8–11]. Approximately 6,000 kidney transplants per year are being carried out in Brazil and have been consolidated as the largest program funded by a public health system in the world. The Brazilian Transplant Registry, published annually, presents information about the transplantation activity in the country and has published data on patient and graft survival in up to 7 years of follow-up [12]. More detailed information, however, about the events responsible for the losses and deaths that have an impact on patient survival and long-term graft survival in our country is scarce.

In addition, previous data have shown that extending the follow-up of transplanted patients beyond the time of occurrence of graft failure may reveal the negative impact of the loss of transplantation function on the survival of the previously transplanted patient [13]. In the United States, between 1988 and 2010, the absolute number of dialysis patients due to renal transplant failure increased by more than 50%, and in 2013, 14% of the patients listed for kidney transplantation had started dialysis due to prior transplant failure, and this type of receptor represented 11.5% of the total number of transplants performed that same year [14, 15]. It is known that graft loss increases by three times the mortality risk when compared with patients with a functioning kidney, and it is estimated that the patient's survival 5 years after graft loss is less than 40% [16, 17]. To date, little is known about the outcomes of kidney transplant patients after return to dialysis, especially in Brazil.

Thus, the objective of this study, carried out in a single Brazilian center, was to evaluate the distribution of the causes of death and graft failure, in a categorized way, through a managed and hierarchical system of information, as well as the incidence of clinical outcomes of patients who return to dialysis after kidney graft failure.

## 2. Methodology

### 2.1. Study Design

This is a retrospective cohort study carried out with kidney transplant patients between 2002 and 2015, in the transplantation program of Hospital Israelita Albert Einstein (Israeli Hospital Albert Einstein), located in the city of São Paulo, Brazil. The city of São Paulo is the capital of São Paulo state and it is the most populous city and state in the country. The health service where the study was conducted is a private hospital that provides philanthropic services through a Ministry of Health funding program called PROADI-SUS. The project was approved by the local ethics committee.

The initial data were extracted during the years 2016 and 2017 from an integrated hospital management system that performs the routine collection of health data from local programs. Thus, all patients included had the possibility of exposure to the risk for at least one consecutive year after transplantation. Patients of any age at the time of transplantation were eligible for this cohort, including transplants performed with pediatric recipients, all of whom had undergone kidney transplantation from a living or deceased donor in our service. The exclusion criteria were as follows: kidney transplant recipients combined with another solid organ (simultaneous with the pancreas, liver, or heart) and transplants performed outside the PROADI-SUS program, that is, those who had supplementary or private medicine as a source of funding, since their follow-up after transplantation was performed independently of the program mentioned above.

### 2.2. Variables and Outcomes

The demographic variables extracted were age, date of transplantation, sex, ethnicity, and donor type. The outcomes considered were graft loss, defined as the need for the permanent return to dialysis after transplantation, death, and loss of follow-up. The events of graft loss or death that occurred in the service itself were identified through the hospital management system. To identify the events that occurred in another health service or outside the health system (in the case of death), the outpatient returns of all patients were monitored by a nurse (author: PRB) responsible for information management (IMN). Through this monitoring, all patients were contacted by phone when they did not attend the consultations for more than 3 months between two consecutive visits. In cases of patients with less than 1 year of transplantation, the contacts were performed when the outpatient return period was not in accordance with the medical conduct indicated at the last visit or by the criterion of returns defined in the institution's care protocol, as described in Table 1.

### 2.3. Categorization of Deaths and Losses

Graft loss was classified in the following groups: primary nonfunction, when the graft lost occurred before function recovery; early death, when patients died after transplant surgery, but before function recovery; death with functioning graft; and graft failure. Deaths were classified into two groups: those that occurred after the patient had renal graft function (i.e., death with the functioning kidney) and those that occurred after the surgical procedure of the transplant, without the recipient having had renal graft function (i.e., early death). The causes of death with the functioning kidney were categorized as infection, cardiovascular disease, neoplasia, or others. Cardiovascular disease was indicated as the cause of the event in both cases when it was the immediate cause of death and when it was the root cause. For the early complications, events that occurred in the 28 days following the transplant surgery were considered in the study, in those which a surgical or clinical complication was the root cause, among patients without graft function.

Graft failure was categorized into thrombosis, acute rejection, chronic rejection, recurrence, or others. Regarding thrombosis, patients with arterial or venous renal thrombosis of the renal graft of mechanical cause were included in the study. All graftectomies of thrombosed grafts were evaluated by pathological anatomy. Those with evidence of an immune-mediated vascular injury (hyperacute or accelerated rejection) were categorized as acute rejection. In the chronic rejection category, cases of loss due to interstitial fibrosis and tubular atrophy, or with evidence of chronic cell-mediated or antibody-mediated rejection, and cases of transplant glomerulopathy were included [18]. Cases which in previous classifications were denominated by the generic term “chronic nephropathy” of the graft were also included here [19].

To minimize the risk of classification bias, the IMN (author name: PRB) presented the event summary to the attending physician responsible for the clinical follow-up of the respective patient (author names: EJT, EFA, LRM, RC, LMMBP, and APB), and the physician indicated one of the categories described above. All categorizations indicated by the attending physicians were reviewed by a single supervising physician (LRM). In the case of disagreement, the records were reviewed, and when there was no consensus, the event was classified as being of unknown etiology.

### 2.4. Identification of Outcomes after Graft Failure

For the patients evaluated with graft failure, the identification of the outcome after the return to dialysis was performed by the IMN (author name: PRB) through follow-up of the evolution of the medical appointments in the program itself, while the patient maintained a follow-up with the medical team. After the loss of clinical follow-up in the transplantation program, the information was monitored through a phone call to the patient, guardian, dialysis clinic, and/or center for the notification, collection, and distribution of organs. Postfailure outcomes were classified as maintenance on dialysis, retransplantation, or death. In cases of death, a copy of the official death certificate was requested for date verification. Due to the inconsistency in completing the field of cause of death in the certificates of these patients, the cause of death was not considered for this analysis.

### 2.5. Statistical Analysis

All transplants in the period that met the eligibility criteria and did not present exclusion criteria were considered for the cohort; therefore, no sample number calculation was performed. The age variable was summarized as the mean and standard deviation because it presented a normal distribution, and for categorization, a multiple of 10 closest to the mean was used: 40 years. The follow-up measures had a nonnormal distribution, so they were summarized as medians, with the interquartile ranges (25%, 75%) as a measure of dispersion. Categorical variables were summarized as percentages. The times to the occurrence of events were compared using the nonparametric Mann-Whitney test, and in the case of comparisons between three groups, an ANOVA analysis was used. Survival and cumulative incidence curves were calculated using the Kaplan-Meier method, and comparisons were made using the log-rank or Breslow-Wilcoxon test. Statistical analyses were performed using SPSS® 25 (IBM, New York, USA), and the graphs were constructed in GraphPad Prism 7 (GraphPad Software, La Jolla, USA). Statistical significance was defined as* p*<0.05, considering a 95% confidence interval.

## 3. Results

**Figure 1:**
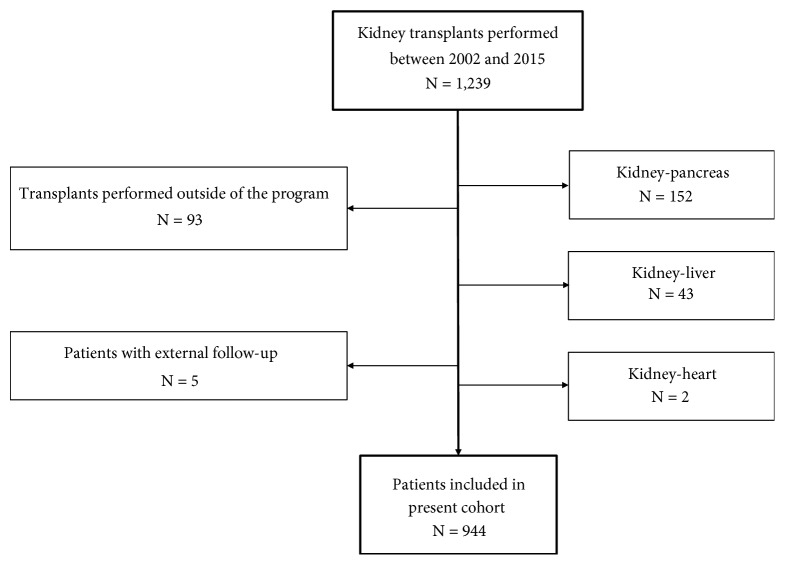
Flow diagram with patients included in this cohort.

Between 2002 and 2015, 1,239 transplants were performed in our center. However, the following transplanted patients were excluded: 197 patients whose kidney transplants were combined with another solid organ (2 heart-kidney, 43 liver-kidney, and 152 pancreas-kidney), 93 transplants performed outside the program (with private or supplementary medicine as a funding source), and five patients who were transplanted through the program but completed follow-up outside the program. Thus, a total of 944 patients were included in the study (Figure 1).

There was a predominance of male recipients (57.4%, n=542) and of the white ethnicity (68.8%, n=642), averaging 43.5±14.1 years, and 54.2% received grafts from deceased donors. At the time of transplantation, only 2.2% (n=21) of the patients were less than 18 years old, 38.9% (n=367) were between 18 and less than 40 years old, 53.4% (n=504) were between 40 and 65 years old, and 5.5% (n=52) were 65 years old or older. The follow-up time was 68.1 (range 31.6-112.4) months. In this period, 217 grafts were lost (23.0%): 92 were due to previously functioning graft failure (42.4%), 92 were due to death with the functioning kidney (42.4%), 23 were due to the primary nonfunction (10.6%), and 10 patients died due to surgical or early clinical complications without having presented renal function (4.6%). Among patients with a primary absence of function, three had acute tubular necrosis (ATN) without the recovery of function within 3 months of follow-up after transplantation (1.4%) and therefore remained on dialysis, and 20 had arterial or venous thrombosis of mechanical origin (9.2%).

### 3.1. Assessment of Causes of Death

**Figure 2:**
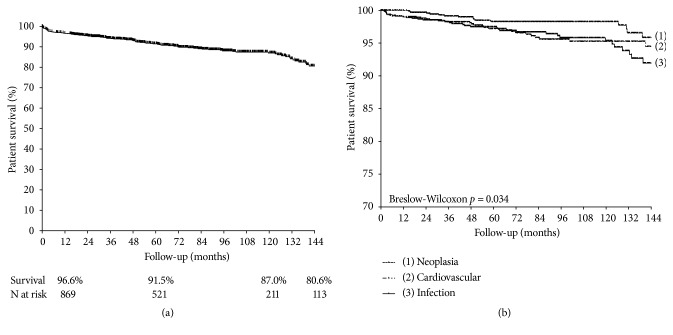
Patient survival (a) and survival curves according to the three main categories of events (b).

The crude mortality rate was 10.8% (n=102), with a mean occurrence time of 35.2 (range 5.8-73.5) months after transplantation. One-, 5-, and 10-year patient survival were 96.6%, 91.5%, and 87.0%, respectively (Figure 2(a)). Infection was the cause of death in 35.3% (n=36) of the cases, followed by cardiovascular events (30.4%, n=31), neoplasia (15.7%, n=16), and death due to early complications (9.8%, n=10). The other causes together accounted for 8.8% (n=9). Of the early deaths, six were due to surgical complications: three due to hemorrhagic shock and three due to distributive shock. The other four occurred as a consequence of clinical complications: two patients with a mycotic aneurysm due to* Staphylococcus aureus* transmitted by the same donor, one due to a coagulation disorder, and another due to hemophagocytic syndrome. The distribution of the frequency of deaths according to the cause is summarized in Table 2. The time between transplantation and the occurrence of the event did not differ among the three main causes of death (*p*=0.47): infection at a mean of 44.1 (range 12.3-113.5) months, cardiovascular events at a mean of 47.4 (range 5.2-71.7) months, and neoplasia at a mean of 45.2 (range 24.8-109.6) months.  Figure 2(b) shows the patient survival curves according to these three main categories of events.

### 3.2. Evaluation of Causes of Graft Loss, Censored for Death

**Figure 3:**
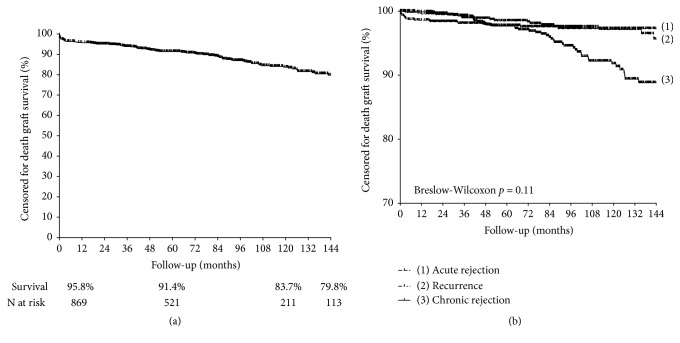
Graft survival censored for death (a) and survival curves according to the three main categories of events (b).

The rate of graft loss, censored for death, was 12.2% (n=115), with a mean occurrence time of 38.1 (range 2.3-84.9) months. So, at the end of 1, 5, and 10 years the death-censored graft survival was 95.8%, 91.4%, and 83.7%, respectively (Figure 3(a)). Chronic rejection was the cause of loss in 40.0% (n=46) of the cases, followed by acute rejection (18.3%, n=21), thrombosis of mechanical etiology (17.4%, n=20), and recurrence of the disease (16.5%, n=19). All other causes combined accounted for 7.8% (n=9). Details about causes of graft loss are depicted in Table 2.

Disregarding surgical causes due to their immediate nature, loss due to acute rejection was the earliest occurrence, at a mean of 3.2 (range 0.76-32.3) months (*p*<0.001, compared with recurrence and chronic rejection), with 61.9% of the events occurring in the first year of transplantation. Loss due to recurrence occurred at a mean of 50.4 (range 20.0-86.5) months, with 21.0% of the events occurring in the first year, 37.1% between the first and fifth year, and 41.9% after the fifth year. Finally, chronic rejection loss was later than recurrence, occurring at a mean of 83.0 (range 42.9-105.8) months, but this difference was not significant (*p*=0.10). Only 34.9% of chronic rejection losses occurred within the first 5 years after transplantation.  Figure 3(b) shows the death-censored graft survival curves, according to these three main categories of events.

### 3.3. Outcomes in Patients with Functioning Kidneys

Excluding the losses and deaths in patients whose graft never had function (primary nonfunction, n=23; early death, n=10), death with the functioning kidney was the main cause of loss (50.0%, n=92), followed by chronic rejection (25.0%, n=46), acute rejection (11.4%, n=21), and recurrence (10.3%, n=19). The other causes accounted for 3.3% of the losses (n=6). Death with a functioning kidney occurred earlier than recurrence and chronic rejection (*p*=0.008), with a mean occurrence after transplantation of 45.9 (range 14.8-81.8) months and with 22.8% of the events occurring in the first year, 21.8% between the first and third year, 18.5% between the third and fifth year, and 36.9% after the fifth year of transplantation.

Graft survival uncensored for death at the end of 1, 5, and 10 years was 92.4%, 83.4%, and 72.7%, respectively.  Table 3 summarizes these rates according to the type of donor (living vs. deceased), with the time the transplant was performed, by gender, age, and ethnicity of the recipient. Over a decade, graft survival was significantly higher among living donor recipients (*p*<0.001) and among those who received a transplantation under the age of 40 (*p*<0.001). There was a trend toward a better result among black recipients (*p*=0.07), and there was no difference regarding gender. Within 5 years of follow-up, no differences were observed according to the period in which the transplantation was performed.

### 3.4. Outcomes after Graft Loss

**Figure 4:**
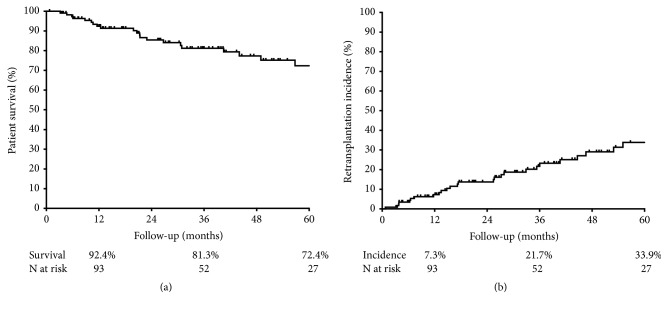
Outcomes after kidney graft failure: patient survival (a) and accumulated incidence of retransplantation (b).

The status of the patients who lost the graft was followed up for a mean of 30.9 (range 13.0-53.4) months after returning to dialysis. Among these, 25 (21.7%) patients died, at a mean of 21.4 (range 10.4-46.5) months after returning to dialysis. One-, 3-, and 5-year patient survival after the return to dialysis were 92.4%, 81.3%, and 72.4%, respectively (Figure 4(a)). Thirty patients (26.1%) underwent a retransplantation, at a mean of 25.5 (range 10.6-45.1) months after the loss of the previous graft. The cumulative incidence of retransplantation at the end of 1, 3, and 5 years was 7.3%, 21.7%, and 33.9%, respectively (Figure 4(b)). Among the 60 patients (52.2%) who remained on dialysis, still alive and not transplanted again until the final period of data collection, the follow-up time after graft failure was 39.3 (range 20.9-71.8) months, and 41.7% (n=25) were enrolled on the list and were active in the transplant program of our service, 33.3% (n=20) were enrolled and active on another list, and the others were not registered.

## 4. Discussion

According to the Brazilian Transplant Registry, in 2017 there were 185 active teams for kidney transplantation in the country, and 5,492 transplants were notified. Considering the vertical nature of the kidney transplantation program, since between 17% and 20% of all kidney transplants are performed in a single center, the average annual number of transplants performed at the center evaluated in the present study (between 60 and 70 transplants per year) was slightly above the national average. Therefore, it is considered that the long-term outcomes of a Brazilian, single-center cohort with average transplantation activity are presented here. Initially, we observed that patient and graft survival were comparable with results found in United States and Australian registry data, and deceased donor outcomes were slightly above the average of the state of São Paulo, where the study hospital is located, especially after 5 years of follow-up. We summarized the data used as a basis for comparison in Table 4 [12, 20–22].

We presented the frequencies of events responsible for deaths and graft losses, from a managed and hierarchical system, in addition to efforts to identify all the events that occurred outside the operation in the transplant center. It was observed that this strategy guaranteed the classification of 93% of the deaths and 99.1% of the losses. In an American study evaluating the outcomes of 1,317 consecutive kidney transplants, El-Zoghby et al. were unable to determine the cause of death in 31.2% of the patients evaluated (a percentage 4.6 times higher than ours), and the follow-up of the cohort was not available [11]. In some countries, long-term follow-up of transplanted patients does not occur in the transplant center and may be performed by nonspecialized teams [10]. Brazil has the largest public transplantation program in the world, with 90% of the kidney transplants being funded by the government through the public health system. One of the characteristics is that the clinical follow-up, as well as the management of late complications, is performed in the transplantation center itself. Even with these characteristics, based on previously published data, it was not possible to classify the cause of death in 17.8% of the cases [23], and this percentage is 2.6 times higher than those found in the present study. We attribute this difference to the methodology used to identify and classify events.

The pattern of distribution of causes of death with a similar frequency of events of infectious and cardiovascular origin differs from previously published Brazilian data [23], where the frequency of death due to infectious disease was observed to be approximately 25% higher than those due to cardiovascular events. To contextualize this finding, the authors considered two important arguments. The most relevant of these was the epidemiological context in which our country is inserted, with high prevalence of infectious diseases when compared with developed countries [24–27]. In addition, when we look at American or European database results, transplanted patients are on average older and more frequently present with chronic kidney disease due to diabetes, two important risk factors for cardiovascular outcomes [20, 28]. Our study has the limitation of not having explored sociodemographic and clinical variables, which could justify the difference in the distribution of causes of death. However, it sheds light on a methodology of the verification of causes of death, with managed execution, which ensured the identification and the classification of almost all events. It should be considered, therefore, that the frequency of death attributed to unknown causes has been high in similar studies, but with a reduction of this categorization to less than 10%, we did not observe a difference in the frequency of death due to infectious and cardiovascular disease.

When analyzing the combined graft losses, we observed a similar number of grafts lost due to death in a functioning kidney receptor and renal failure in a previously functioning graft. At the beginning of the last decade, accurate data from the Australian records showed that death with the functioning kidney was one of the main causes of graft loss, however, with less impact than chronic graft loss, which was classified at that time as chronic allograft nephropathy [9, 29]. More recently, information published by other major transplant centers has shown that death with the functioning kidney is the main cause of graft loss [11, 23, 30]. It is very likely that this variation in the different publications reflects the demographic and epidemiological characteristics of the transplanted populations, with variable interference in the range of the main causes of mortality, cardiovascular, infectious, and neoplastic diseases, as discussed above [9, 23]. In addition, the follow-up time may interfere with the incidence of each category, since the causes of loss are distributed asymmetrically over time. Although the deaths in our cohort occurred earlier than the losses due to chronic rejection and disease recurrence, they occurred later than those observed in the cohort followed by El-Zoghby et al.: death after 5 years of transplantation: 36.9% vs. 23.9% [11].

As expected, and similar to that previously observed, acute rejection was the cause of earlier graft loss, with more than 60% of events occurring within the first year. However, there was a small impact on the cohort (2.2%), reflecting the improvement in the quality of immunosuppression observed in the last decades and more sensitive techniques of pretransplant immunological evaluation [31–34]. Despite this, it should be considered that late acute rejection loss still accounts for 1/3 of the losses in this category, reflecting other possible variables that have gained considerable interest in the recent transplantation era, such as adherence to immunosuppressive therapy [35, 36]. The other two groups categorized as graft loss, chronic rejection and recurrence of the disease, are often of late occurrence, even when reflecting events of an early nature [11, 29]. Chronic rejection is the final route of recurrent episodes of acute cell rejection [37, 38] or the glomerular or tubulointerstitial manifestation of antibody-mediated events [39, 40], whereas loss due to recurrence reflects the impact of pathologies of very early occurrences, such as segmental and focal glomerulosclerosis, as well as later ones such as membranous or membranous-proliferative glomerulopathy [29, 41].

In an exploratory way, we compared the graft survival results, uncensored for death, according to the variables that are managed in the program. As expected, the results were significantly better in living donor recipients and in those transplanted at a younger age. The best evolution is known to be in living donor recipients, and the reasons for this vast difference have been widely discussed, especially the shorter waiting time for transplantation and, therefore, fewer morbidities associated with the evolution of chronic kidney disease, better compatibility, and, mainly, the quality of the organ [8, 10, 11]. Likewise, higher evolution in transplants performed in young recipients is expected in relation to those of an older age, especially when the outcomes evaluated include mortality [42, 43]. Aging is related to the increase in the risk of dying, mainly due to the addition of morbidities such as hypertension, diabetes, other factors for cardiovascular disease, and neoplasia [43, 44].

The clinical outcomes of kidney transplantation do not end when the transplanted patient has the complete and irreversible failure of graft function. By prolonging the follow-up of patients beyond the period of graft failure, it is observed that the return to dialysis has a significant negative impact. The mortality of patients who lose graft function increases 2 to 3 times after the return to dialysis [13, 17], ranging from 25% to 50% [16]. In our cohort, we observed a mortality of 21.7% after graft failure, occurring on average 2 years after the return to dialysis. In addition to the limitation of not investigating clinical variables, it was not possible to identify the causes of death after graft failure, considering that most of these outcomes occurred in another health unit and, in our country, completing the official documents that attest the cause of death is a weak process.

Another variable with interference in the evolution of the patient after graft failure is the performance of a new transplant. When transplanted patients who return to dialysis are compared with those who have never been transplanted and are on the waiting list, it is observed that the risk of dying is three times higher among those who have already been transplanted [13]. Although the results after retransplantation are inferior compared with the first transplant, some data suggest that a new transplant is the best option of renal replacement therapy for patients who present prior transplant failure and is, therefore, a strategy capable of decreasing mortality after the loss of the graft. In 2013, 11.5% of patients transplanted in the United States underwent a retransplantation [14]. In our service, this rate varies between 10% and 20%. In our cohort, we observed a high index of reenrolled and active patients on the waiting list for a new transplant, most of them being followed up in the service itself. This characteristic may justify the high rate of retransplantation observed, 26.1%, which is higher than the crude mortality rate.

Some limitations should be highlighted in our study. Like any observational study, there is a threat to the validation of results, especially since we did not include detailed demographic information or details of the clinical evolution, such as the type of immunosuppression or details of morbidity. Despite the great effort to categorize the causes of death and loss, which generated a low index of unclassified causes of death, the chronic losses were categorized as chronic rejection, which occasioned a risk of classification bias. Finally, the study was limited in evaluating details about the follow-up of patients after graft failure. We have considered only the hard outcome such as death, retransplantation, or remaining on dialysis, without exploring more details or the variables associated with the chance of retransplantation and risk of death.

## 5. Conclusion

In conclusion, this analysis has shown that the causes of loss and death are not very different from other data available in the literature, with one of the most relevant results being the demonstration that the active search for information in a managed and hierarchical way is able to clarify most of the causes of death and loss. In addition, this is the first Brazilian study to identify the outcomes of transplanted patients after graft failure. This information is of the utmost importance to the public health authorities involved in the planning of substitute renal therapies, given the increasing number of patients who return to dialysis after the failure of a previous renal graft and especially since, in the global context, Brazil has one of the largest public renal replacement therapy programs and the largest public transplant program.

## Figures and Tables

**Table 1:** Frequency of outpatient medical visits according to institution's care protocol.

Time after transplantation	Frequency
1^st^ month	2 returns weekly
2^nd^ month	1 return weekly
3^rd⁡^ month	1 return every 15 days
4^th^ and 5^th^ month	1 return every 3 weeks
From 6^th^ to 12^th^ month	1 return monthly
From 12^th^ to 24^th^ month	1 return every 2 months
After 24^th^ month	1 return every 3 months

**Table 2:** Causes of death and graft failure after transplantation and their impact on the total number of patients.

Event	N	% to total events	% to initial cohort
Death	102	100	10.8
Infection	36	35.3	3.8
Cardiovascular	31	30.4	3.3
Neoplasia	16	15.7	1.7
Early complications^*∗*^	10	9.8	1.1
Surgical	6	5.9	0.6
Clinical	4	3.9	0.4
Other	9	8.8	0.9
Unknown	7	6.8	0.7
Gunshot wounds	1	1.0	0.1
Cirrhosis	1	1.0	0.1

Losses	115	100	12.2

Chronic rejection	46	40.0	4.9
Acute rejection	21	18.3	2.2
Thrombosis	20	17.4	2.1
Recurrence	19	16.5	2.0
Other	9	7.8	0.9
Primary nonfunction^#^	3	2.6	0.3
Pyelonephritis	2	1.7	0.2
Unknown	1	0.9	0.1
Biopsy complication^§^	1	0.9	0.1
Cirrhosis	1	0.9	0.1
Polyoma nephropathy	1	0.9	0.1

^*∗*^Early complications: events that occurred in the 28 days following the transplant surgery, without the recipient having had renal graft function.

^#^Primary nonfunction: patients who presented acute tubular necrosis after transplantation without the recovery of function within 3 months of follow-up.

^§^Loss occurred after a graft biopsy complication as an adverse major event.

**Table 3:** Graft survival uncensored for death.

Variables	Baseline	1 year	5 years	10 years	*p*
Donor	Living	95.4%	88.6%	79.2%	
	(432)	(409)	(275)	(126)	
	Deceased	89.8%	78.8%	66.5%	
	(512)	(459)	(245)	(85)	<0.001^#^

Time	2002-2007	91.2%	83.4%	73.2%	
	(333)	(302)	(274)	(201)	
	2008-2011	92.7%	81.2%	-	
	(288)	(266)	(233)		
	2012-2015	93.2%	83.7%	-	
	(323)	(300)	(13)		0.42^*∗*^

Gender	Female	93.0%	86.4%	76.0%	
	(402)	(373)	(232)	(104)	
	Male	91.9%	81.1%	70.7%	
	(542)	(495)	(288)	(106)	0.13^#^

Age	< 40 years	93.9%	87.8%	78.6%	
	(411)	(382)	(244)	(115)	
	≥ 40 years	91.2%	79.9%	67.9%	
	(533)	(486)	(281)	(95)	<0.001^#^

Ethnicity	Black	95.2%	89.6%	78.9%	
	(83)	(79)	(46)	(16)	
	Nonblack	92.1%	82.8%	72.1%	
	(861)	(789)	(474)	(94)	0.07^#^

Numbers in parentheses: number at risk.

^#^ *p* performed by the log-rank test.

^*∗*^ *p* performed by the Breslow test.

**Table 4:** Patient and graft survival used as a basis for comparison with the results observed in the present study.

	*Patient survival*			*Graft survival*		
	1 year	5 years	10 years	1 year	5 years	10 years
SES-SP						
Living	-	-	-	-	-	-
Deceased	89.5	80.0	66.8	86.8	73.8	60.6

RBT						
Living	97.0	94.0	-	93.0	86.0	-
Deceased	92.0	86.0	-	84.0	73.0	-

ANZDATA						
Living	99.0	95.0	89.0	97.0	90.0	76.0
Deceased	97.0	90.0	75.0	92.0	81.0	60.0

OPTN						
Living	98.8	92.0	-	97.5	85.6	-
Deceased	96.2	83.1	-	93.2	74.4	-

Present study						
Living	98.8	95,4	92.6	95.4	88.6	79.2
Deceased	94.8	87.9	81.4	89.8	78.8	66.5

SES-SP: results in state of São Paulo according to official government data, accessed in October 2017, considering all kidney transplants performed between 2002 and 2015 [21].

RBT: Brazilian Transplant Registry, considering only medical groups that have informed 100% of their results (79% of the total estimated) in 2016 [12].

ANZDATA: transplants performed between 2005 and 2009. 39th Annual Report. ANZDATA Registry [22].

OPTN: to 1-year survival; we have considered transplants performed between 2012 and 2015; to 5-year survival; we have considered transplants performed between 2008 and 2011 [20].

## Data Availability

The spreadsheet containing all data was built using data available in secondary fount of information as medical records and management's data bank. This spreadsheet used to support the findings of this study is available from the corresponding author upon request and may be released upon application to the Comitê de Ética em Pesquisa, Hospital Israelita Albert Einstein, which can be contacted at cep@einstein.br.
